# Association between skeletal muscle attenuation and gastroesophageal reflux disease: A health check-up cohort study

**DOI:** 10.1038/s41598-019-56702-6

**Published:** 2019-12-27

**Authors:** Young Min Kim, Jie-Hyun Kim, Su Jung Baik, Da Hyun Jung, Jae Jun Park, Young Hoon Youn, Hyojin Park

**Affiliations:** 1 Department of Internal Medicine, Gangnam Severance Hospital, Yonsei University College of Medicine, Seoul, Korea; 2 Department of Healthcare Research Team, Health Promotion Center, Gangnam Severance Hospital, Yonsei University College of Medicine, Seoul, Korea

**Keywords:** Gastro-oesophageal reflux disease, Oesophagus

## Abstract

Sarcopenia is defined as skeletal muscle attenuation and has an association with metabolic syndrome. Metabolic syndrome, which includes obesity, is one of known predictive factors for gastroesophageal reflux disease (GERD). This study aimed to elucidate the association between sarcopenia and GERD. We retrospectively reviewed electronic medical records of 8,218 patients who were performed an upper gastrointestinal endoscopy at check-up center of the Gangnam Severance Hospital. GERD was diagnosed by endoscopic findings. Erosive reflux disease (ERD) included Barrett's esophagus and reflux esophagitis, with the exception of minimal change esophagitis. Sarcopenia was defined by appendicular skeletal muscle (skeletal muscle in the upper and lower limbs). Sarcopenic obesity was defined as the presence of both sarcopenia and obesity. Associations between sarcopenia and GERD, as well as between sarcopenic obesity and ERD, were analyzed. A total of 3,414 patients were diagnosed with GERD, and 574 (16.8%) had sarcopenia. Sarcopenia was independent predictive factor for GERD (odds ratio [OR] = 1.170, 95% confidence interval [CI]: 1.016–1.346, *P* = 0.029). In addition, male sex, smoking, alcohol, and diet, including sweets and fatty food, had a significant association with GERD. A total of 1,423 (17.3%) of 8,218 patients were diagnosed with ERD, and 302 (21.2%) had sarcopenia. Male sex, smoking, and fatty food consumption had a significant association with ERD. Moreover, sarcopenia (OR = 1.215, 95% CI: 1.019–1.449, *P* = 0.030), obesity (OR = 1.343, 95% CI: 1.163–1.552, *P* < 0.001), and sarcopenic obesity (OR = 1.406, 95% CI: 1.195–1.654, *P* < 0.001) were independent predictive factors for ERD. Sarcopenia is associated with GERD, and sarcopenic obesity may be predictive factor for ERD.

## Introduction

Sarcopenia is progressive skeletal muscle attenuation that occurs as a consequence of the aging process. Baumgartner *et al*. first defined sarcopenia by appendicular skeletal muscle mass (skeletal muscle mass in the upper and lower limbs, ASM, kg)/height^2^ (m^2^) in 1998^1^. Although there have been several different suggestive definitions of sarcopenia^2–5^, consensus definition for clinical research and practice is scarce^3,6^. The decline in muscle mass that begins around the fourth decade of life is approximately 25–30% during lifetimes^7,8^. With an increase in the world's advanced age population, the prevalence of sarcopenia has been increasing but has a wide variation according to the definition, method of measurement for muscle mass, age, race, and sex^9,10^. Sarcopenia is considered as a major health concern, because it is associated with physical disability, low quality of life, and mortality^3,11^. Previous studies have shown that sarcopenia has an association with metabolic syndrome. Since skeletal muscle is the main tissue for the insulin-dependent glucose uptake, sarcopenia is associated with the systemic insulin resistance that is a component of metabolic syndrome^12,13^. One study reported that sarcopenic obesity, which is the presence of both sarco penia and obesity that are associated with metabolic syndrome, has a greater impact on metabolic risk and health outcome^6^.

Gastroesophageal reflux disease (GERD) is a more common disease in Western populations than in Asian populations. A GERD prevalence of approximately 10–20% was identified in Western populations and was less than 5% in Asian populations^14^. However, in Asia, the prevalence of GERD has been increasing dramatically over recent decades because of Westernized diets, extended life expectancy, and increased eradication rate of *Helicobacter pylori*^15^. Previous studies reported that metabolic syndrome and insulin resistance are one of the risk factors for GERD. One study reported that an increased triglyceride (TG) level, which is associated with metabolic syndrome, is an independent risk factor for GERD^16^. Interleukin (IL)−6 is a cytokine that stimulates hepatic TG secretion, has a key role in insulin resistance^17,18^, and is increased in Barrett's esophagus^19^.

Since both sarcopenia and GERD are interlinked with metabolic syndrome, we developed a hypothesis that sarcopenia is associated with GERD. However, the data demonstrating this relationship have been scarce.

The aim of our study was to investigate the predictive factors for GERD, including erosive reflux disease (ERD), with a focus on sarcopenia and sarcopenic obesity among patients who were performed a health check-up program. In addition, we evaluated the correlation between skeletal muscle index (SMI) as an estimator of sarcopenia and body mass index (BMI) that estimates obesity in patients with sarcopenic obesity.

## Method

### Study design and population

**Figure 1:**
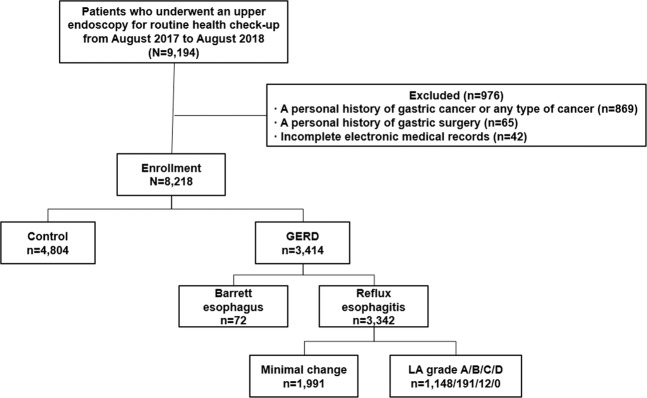
Flow chart of the enrolled patients.

A total of 9,194 patients who underwent an upper gastrointestinal endoscopy at the Health Promotion Center of the Gangnam Severance Hospital, Seoul, South Korea as part of a routine health check-up were considered for this cross-sectional and retrospective study. We reviewed the patients’ electronic medical records from August 2017 to August 2018, when is able to measure SMI in our check-up center. The exclusion criteria were as follows: (1) a personal history of malignancy including gastric cancer (n = 869), (2) a personal history of stomach operation, such as a resection of the stomach or gastrectomy (n = 65), and (3) incomplete electronic medical records (n = 42). As a result, 8,218 patients were enrolled in our study. All enrolled patients were Korean. Figure 1 shows a flow chart of the enrolled patients.

The study protocol conformed to the ethical guidelines of the World Medical Association Declaration of Helsinki and was approved by the Institutional Review Board of Gangnam Severance Hospital (IRB No: 3-2018-0102). Since this study was a retrospective analysis of existing administrative and clinical data, informed consent was not required.

### Anthropometric measurements

We measured anthropometric parameters in all enrolled patients. Body weight and height were measured to the nearest 0.1 kg and 0.1 cm, respectively with the patients barefoot and putting on light-weight clothing. Multi-frequency bioelectrical impedance analysis (ACCUNIQ BC 720; SELVAS healthcare, Korea) was used to measure skeletal muscle mass, which is considered an appropriate device to dual-energy X-ray absorptiometry^20^. ASM was calculated as the total of appendicular lean mass of both arms and legs^21^. BMI was defined as body weight (kg) divided by height squared (m^2^).

We adopted the definition of sarcopenia developed by Janssen *et al*.^2^; they used the SMI to evaluate the prevalence of sarcopenia and defined sarcopenia as a SMI less than one standard deviation below the sex-specific mean for healthy subjects aged 20 to 39 years. SMI was calculated by ASM as a percentage of body weight. In our study, the mean SMI of healthy adults was 32.9 ± 3.6% and 29.8 ± 3.1% in males and females, respectively. In our study, cutoff value for sarcopenia was 29.3% in males and 26.7% in females. Therefore, sarcopenia was diagnosed as SMI ≤ 29.3% in males and ≤ 26.7% in females. We used the definition of obesity with Asia-Pacific criteria^22^, which is a BMI ≥ 25 kg/m^2^. Sarcopenic obesity is the combination of sarcopenia and obesity in our study.

### Questionnaire

All patients who underwent our health check-up program were asked to fill out a questionnaire. Smoking history, alcohol history, combined diabetes mellitus (DM), and diet, which included rapid intake, irregular intake, sweets, fatty foods, caffeinated drinks, and spicy foods, were included in the questionnaire. Rapid intake meant that it took less than 15 minutes to have one meal. Patients were asked to select “yes” or “no” for irregular intake. For the diet, which included sweets, fatty foods, caffeinated drinks, and spicy foods, a patient selected “yes” if they consumed that particular diet item over six times within seven days.

### Upper gastrointestinal endoscopy

All enrolled patients were performed upper gastrointestinal endoscopic examination using an endoscope (GIF-H260; Olympus Medical Systems, Tokyo, Japan) with an electronic endoscopic system (EVIS LUCERA; Olympus Medical Systems). The diagnosis of GERD was made by endoscopists who had at least 3 years of experience. The severity of reflux esophagitis was graded according to the Los Angeles (LA) classification^23^. The GERD group was comprised of patients who had non-erosive reflux disease and ERD. Patients who had minimal change esophagitis were classified as non-erosive reflux disease, and patients with obvious evidence of esophageal mucosal injury on the endoscopy, such as Barrett's esophagus and LA grade A to D reflux esophagitis, were classified as ERD-(1) group. And LA grade B to D reflux esophagitis was classified as ERD-(2) group^24^.

### Statistical analysis

Continuous variable values were reported as the mean ± standard deviation. The t-test or Wilcoxon rank-sum test was performed to compare continuous variables between the groups. Categorical variable values were reported as the number and percentage, and we used the chi-square test or Fisher’s exact test to compare categorical variables.

The Pearson's correlation analysis was used to evaluate the correlation between two continuous variables. Moreover, multivariate Cox regression analysis was performed to investigate the predictive factors for GERD and ERD. Since sarcopenic obesity is a combination of sarcopenia and obesity, two multivariate analyses were performed. We used SPSS version 23.0 (IBM Corp., Armonk, NY, USA) for statistical analysis. Statistical significance was determined by a two-tailed *P*-value < 0.05.

## Results

### Baseline characteristics

Baseline characteristics of the study population are showed in Table 1. Of the 8,218 patients, 4,622 (56.2%) were men, and the mean age was 48.9 ± 11.6 years. The average BMI was 23.9 ± 3.4 kg/m^2^. The mean SMI was 32.2 ± 2.7% in male and 28.8 ± 3.0% in female. A total of 15.1% and 13.4% of the patients were diagnosed with sarcopenia and sarcopenic obesity, respectively. The male had a higher prevalence of sarcopenia (18.7% male vs 10.5% female) and sarcopenic obesity (17.2% male vs 8.5% female) than female.

**Table 1:** Baseline characteristics of the study population.

Characteristics	All patients (N = 8,218)
Age (years, mean ± SD)	48.9 ± 11.6
Male (n, %)	4,622 (56.2)
Height (cm, mean ± SD)	166.6 ± 8.5
Weight (kg, mean ± SD)	66.6 ± 12.9
BMI (kg/m^2^, mean ± SD)	23.9 ± 3.4
Obesity (n, %)	2,799 (34.1)
SMI (%, mean ± SD)	
Male	32.2 ± 2.7
Female	28.8 ± 3.0
Current smoker (n, %)	1,470 (17.9)
Alcohol history (n, %)	5,482 (66.7)
Combined DM (n, %)	1,190 (14.5)
Sarcopenia (n, %)	1,242 (15.1)
Male	864/4,622 (18.7)
Female	378/3,596 (10.5)
Sarcopenic obesity (n, %)	1103 (13.4)
Male	797/4,622 (17.2)
Female	306/3,596 (8.5)
Diet style (n, %)	
Rapid intake	2,938 (35.8)
Irregular intake	2,406 (29.3)
Sweets	2,343 (28.5)
Fatty foods	1,759 (21.4)
Caffeinated drinks	3,894 (47.4)
Spicy foods	1,139 (13.9)
Endoscopic finding	
Barrett’s esophagus	72
Minimal change esophagitis	1,991
Reflux esophagitis LA grade A/B/C/D	1,148/191/12/0

BMI, body mass index; SMI, skeletal muscle index; DM, diabetes mellitus; GERD, gastroesophageal reflux disease.

A total of 3,414 patients were classified as GERD by endoscopic findings. Among these patients, 3,342 had reflux esophagitis, 1,991 had minimal change esophagitis, 1,148 had LA grade A, 191 had LA grade B, and 12 had LA grade C. Moreover, 72 had short-segment Barrett's esophagus.

### Predictive factors for GERD

As a result of the univariate analysis, advanced age, male sex, obesity, smoking, alcohol, sarcopenia, sarcopenic obesity, and diet, which included sweets, fatty foods, and caffeinated drinks, had a significant association with an increased risk of GERD. In the multivariate analysis, sarcopenia was independent predictive factor for GERD (odds ratio [OR] = 1.170, 95% confidence interval [CI]: 1.016–1.346, *P* = 0.029). Moreover, male sex (OR = 1.493, 95% CI: 1.344–1.658, *P* < 0.001), smoking (OR = 1.139, 95% CI: 1.007–1.287, *P* = 0.038), alcohol (OR = 1.121, 95% CI: 1.013–1.241, *P* = 0.027), and diet, which included sweets (OR = 1.132, 95% CI: 1.024–1.251, *P* = 0.015) and fatty foods (OR = 1.212, 95% CI: 1.085–1.354, *P* = 0.001), were independent predictive factors for GERD. However, obesity was not independently associated with GERD in multivariate analysis.

### Predictive factors for ERD

Predictive factors for ERD-(1) group were shown in Tables 2 and 3. As a result of the univariate analysis, advanced age, male sex, obesity, smoking, alcohol, sarcopenia, sarcopenic obesity, and diet, which included fatty foods, caffeinated drinks, and spicy foods, had a significant association with an increased risk of ERD-(1) group (Table 2). Among these factors, sarcopenia (OR = 1.215, 95% CI: 1.019–1.449, *P* = 0.030), obesity (OR = 1.343, 95% CI: 1.163–1.552, *P* < 0.001), and sarcopenic obesity (OR = 1.406, 95% CI: 1.195–1.654, *P* = 0.001) were independent predictive factors for ERD-(1). Moreover, male sex (OR = 2.447, 95% CI: 2.098–2.853, *P* < 0.001), smoking (OR = 1.259, 95% CI: 1.081–1.466, *P* = 0.003), and fatty foods (OR = 1.327, 95% CI: 1.148–1.536, *P* < 0.001) had an independent association with ERD-(1) (Table 3).

**Table 2:** Univariate analysis of the predictive facfstors for ERD-(1) group.

	Univariate analysis		
	Control (n = 4,804)	ERD-(1) (n = 1,423)	*P*-value
Age (years, mean ± SD)	48.1 ± 11.7	49.5 ± 11.5	0.031
Male (n, %)	2,464 (51.3)	1,088 (76.5)	<0.001
Obesity (n, %)	1,542 (32.1)	683 (48.0)	<0.001
Current smoker (n, %)	755 (15.7)	387 (27.2)	<0.001
Alcohol history (n, %)	3,081 (64.1)	1,055 (74.1)	<0.001
Combined DM (n, %)	696 (14.5)	212 (14.9)	0.700
Diet style (n, %)			
Rapid intake	1,754 (36.5)	541 (38.0)	0.301
Irregular intake	397 (27.9)	1,443 (30.0)	0.120
Sweets	382 (26.8)	1,324 (27.6)	0.595
Fatty foods	925 (19.3)	394 (27.7)	<0.001
Caffeinated drinks	2,230 (46.4)	756 (53.1)	<0.001
Spicy foods	655 (13.6)	237 (16.7)	0.004
Sarcopenia (n, %)	668 (13.9)	302 (21.2)	<0.001
Sarcopenic obesity (n, %)	595 (12.4)	274 (19.3)	<0.001

ERD, erosive reflux disease; BMI, body mass index; DM, diabetes mellitus.

ERD-(1) group: consisted of Barrett’s esophagus and LA grade A to D reflux esophagitis.

**Table 3:** Multivariate analysis of the predictive factors for ERD-(1) group.

	Multivariate analysis 1		Multivariate analysis 2	
	OR (95% CI)	*P*-value	OR (95% CI)	*P*-value
Male	2.447 (2.098–2.853)	<0.001	2.629 (2.264–3.052)	<0.001
Current smoker	1.259 (1.081–1.466)	0.003	1.265 (1.087–1.473)	0.002
Alcohol history	1.093 (0.945–1.263)	0.230	1.082 (0.936–1.250)	0.286
Diet style				
Fatty foods	1.327 (1.148–1.536)	<0.001	1.339 (1.158–1.548)	<0.001
Caffeinated drinks	1.040 (0.916–1.180)	0.546	1.051 (0.927–1.193)	0.438
Spicy foods	0.981 (0.824–1.168)	0.829	0.994 (0.835–1.182)	0.944
Sarcopenia	1.215 (1.019–1.449)	0.030	—	—
Obesity	1.343 (1.163–1.552)	<0.001	—	—
Sarcopenic obesity	—	—	1.406 (1.195–1.654)	<0.001

OR, Odds ratio.

ERD-(1) group: consisted of Barrett’s esophagus and LA grade A to D reflux esophagitis.

Multivariate analysis 1: Adjusted for male, smoking, alcohol, diet style, sarcopenia, and obesity. Multivariate analysis 2: Adjusted for male, smoking, alcohol, diet style, and sarcopenic obesity.

Tables 4 and 5 show the predictive factors for ERD-(2) group. As a result of the univariate analysis, male sex, obesity, smoking, alcohol, DM, sarcopenia, sarcopenic obesity, and diet, which included fatty foods and caffeinated drinks had a significant association with an increased risk of ERD-(2) group (Table 4). As a result of the multivariate analysis, male (OR = 2.514, 95% CI: 1.756–3.598, *P* < 0.001), smoking (OR = 1.224, 95% CI: 1.042–1.464, *P* = 0.015), sarcopenia (OR = 1.375, 95% CI: 1.020–1.754, *P* = 0.040), obesity (OR = 2.564, 95% CI: 1.972–3.322, *P* < 0.001), and sarcopenic obesity (OR = 2.020, 95% CI: 1.515–2.703, *P* < 0.001) were independently associated with ERD-(2) group (Table 5).

**Table 4:** Univariate analysis of the predictive factors for ERD-(2) group.

	Univariate analysis		
	Control (n = 4,804)	ERD-(2) (n = 275)	*P*-value
Age (years, mean ± SD)	49.5 ± 11.5	49.0 ± 11.8	0.486
Male (n, %)	2,464 (51.3)	223 (81.1)	<0.001
BMI (kg/m^2^, mean ± SD) 0)	23.7 ± 3.3	26.0 ± 2.9	<0.001
Obesity (n, %)	1,542 (32.1)	171 (62.2)	<0.001
Current smoker (n, %)	755 (15.7)	85 (30.9)	<0.001
Alcohol history (n, %)	3,081 (64.1)	208 (75.6)	<0.001
Combined DM (n, %)	696 (14.5)	53 (19.3)	0.030
Diet style (n, %)			
Rapid intake	1,754 (36.5)	104 (37.8)	0.662
Irregular intake	397 (27.9)	86 (31.3)	0.371
Sweets	382 (26.8)	76 (27.6)	0.732
Fatty foods	925 (19.3)	71 (25.8)	0.008
Caffeinated drinks	2,230 (46.4)	147 (53.5)	0.023
Spicy foods	655 (13.6)	35 (12.7)	0.669
Sarcopenia (n, %)	668 (13.9)	75 (27.3)	<0.001
Sarcopenic obesity (n, %)	595 (12.4)	70 (25.5)	<0.001

ERD, erosive reflux disease; BMI, body mass index; DM, diabetes mellitus.

ERD-(2) group: consisted of Barrett’s esophagus and LA grade B to D reflux esophagitis.

**Table 5:** Multivariate analysis of the predictive factors for ERD-(2) group.

	Multivariate analysis 1		Multivariate analysis 2	
	OR (95% CI)	*P*-value	OR (95% CI)	*P*-value
Male	2.514 (1.756–3.598)	<0.001	3.085 (2.174–4.379)	<0.001
Current smoker	1.224 (1.042–1.464)	0.015	1.256 (1.062–1.488)	0.008
Alcohol history	1.104 (0.812–1.499)	0.528	1.086 (0.800–1.473)	0.599
DM	1.221 (0.889–1.678)	0.217	1.259 (0.918–1.727)	0.152
Diet style				
Fatty foods	1.135 (0.850–1.517)	0.391	1.175 (0.880–1.567)	0.274
Caffeinated drinks	1.002 (0.773–1.287)	0.987	1.025 (0.795–1.319)	0.853
Sarcopenia	1.375 (1.020–1.754)	0.040	—	—
Obesity	2.564 (1.972–3.322)	<0.001	—	—
Sarcopenic obesity	—	—	2.020 (1.515–2.703)	<0.001

OR, Odds ratio; DM, diabetes mellitus.

ERD-(2) group: consisted of Barrett’s esophagus and LA grade B to D reflux esophagitis.

Multivariate analysis 1: Adjusted for male, smoking, alcohol, DM, diet style, sarcopenia, and obesity.

Multivariate analysis 2: Adjusted for male, smoking, alcohol, DM, diet style, and sarcopenic obesity.

### Correlation between SMI and BMI in ERD patients

**Figure 2:**
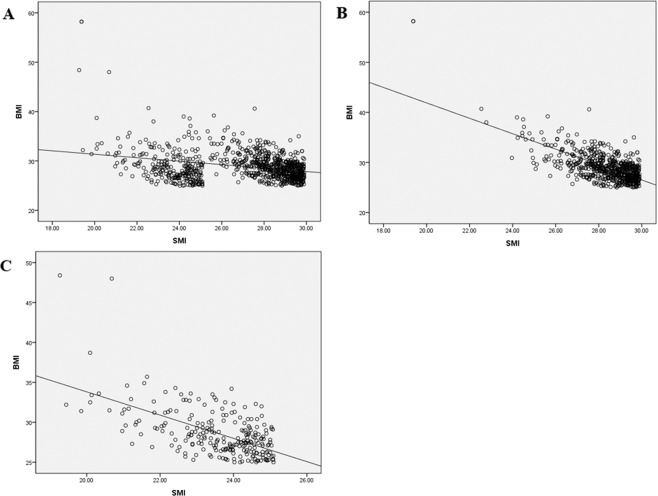
Correlation analysis between body mass index (BMI) and skeletal muscle index (SMI) in patients diagnosed with sarcopenic obesity in the ERD group. (**A**) In both the male and female groups (**B**) In the male group (**C**) In the female group.

Since sarcopenia, obesity, and sarcopenic obesity were independent predictive factors for ERD, we performed an additional analysis to identify the correlation between SMI and BMI. Figure 2A shows the significantly negative correlation between SMI and BMI in the patients who were diagnosed with sarcopenic obesity in the ERD group. (Pearson’s correlation coefficient: −0.301, *P* < 0.001). According to sex, the male group (Pearson’s correlation coefficient: −0.682, *P* < 0.001, Fig. 2B) had a more strongly negative correlation than the female group (Pearson’s correlation coefficient: −0.587, *P* < 0.001, Fig. 2C).

## Discussion

Sarcopenia is a growing health concern because of its negative outcomes from functional decline to death, as well as the increasing worldwide prevalence^25^. There are several known risk factors for GERD, including obesity, structural abnormalities such as hiatal hernia, and specific diets and medications^26^. However, there have beeTablen no studies that link GERD and sarcopenia. Recent studies have reported that sarcopenic obesity has an influence on cardiometabolic diseases^27–31^. In these studies, sarcopenic obesity is a strongly associated with metabolic syndrome and has a higher impact on metabolic diseases and mortality than obesity or sarcopenia alone. To the best of our knowledge, this study is the first to identify an association between GERD and sarcopenia. Notably, the strength of our study is that we performed additional analysis between ERD and the “new concept” of sarcopenic obesity.

In the conclusion of our study, sarcopenia is associated with GERD. This association can be explained by metabolic syndrome. Previous study reported that volume of skeletal muscle fiber is related to the expression of glucose transporter type 4 (GLUT4), which enables the uptake of glucose^32^. Therefore, sarcopenia can induce an insulin intolerance that is a component of metabolic syndrome. There was a vicious cycle between sarcopenia and metabolic syndrome^33^. Muscle mass attenuation from sarcopenia induces lower physical activity that causes visceral f at to increase in the body. Since skeletal muscle is an insulin-response target tissue, patients with sarcopenia develop progressive metabolic syndrome. Although precise mechanisms of association between sarcopenia and GERD are unclear, we guess that sarcopenia might be affect to mechanical barrier, such as function of esophagus and stomach, thereby induce GERD. Moreover, cytokine signaling can be one of mechanisms as mentioned in the section of introduction. Previous study reported that cytokine such as TNF-α, TWEAK, and IL-6 were related to skeletal muscle wasting^34^.

The prevalence of GERD in the Asian population has been reported as generally less than 10%^35^. However, the prevalence of GERD in our study was much higher at 41.5%. In addition, obesity, which is a known potential risk factor for GERD, did not have a statistical significance in our multivariate analysis^36^. These results were due to the following: First, since the questionnaire of our check-up center did not have symptoms for GERD, such as heartburn and regurgitation, non-erosive reflux disease was diagnosed by only endoscopic findings of minimal change esophagitis, which is ambiguous; second, the characteristics of the non-erosive reflux disease group was heterogeneous, and they may have had functional dyspepsia or psychological comorbidities.

Thus, we performed an additional analysis on 1,423 (17.3%) patients who had ERD-(1), which has a more definitive mucosal injury and is more specific for GERD. Sarcopenia, obesity, and sarcopenic obesity were independent predictive factors for ERD-(1) group. Interestingly, sarcopenic obesity (OR 1.406) was more predictive than sarcopenia (OR 1.215) or obesity (OR 1,343) for ERD-(1) group. This result suggests that sarcopenia and obesity may have a synergistic influence on ERD-(1) group. Moreover, there was a significantly negative correlation between SMI and BMI, especially in the male patients. LA grade B to D reflux esophagitis (ERD-(2) group, n = 203) are more specific endoscopic findings for GERD. In this analysis, sarcopenia (OR 1.493), obesity (OR 1.229), and sarcopenic obesity (OR 1.616) also associated with ERD-(2) group. This result overcame our study’s limitation and support conclusion of our study.

As we mentioned above, the prevalence of sarcopenia is different according to race. In the result of meta- analysis, the prevalence of sarcopenia was different according to the method to measure muscle mass^9^. When method BIA used, non-Asian (19%) was higher than Asian (10%) in male, and non-Asian (20%) was higher than Asian (11%) in female. When method DXA used, Asian (9%) was higher than non-Asian (6%) in male, and non-Asian (10%) was higher than Asian in female (6%). Therefore, it is necessary to make consensus diagnostic tool for sarcopenia, and further study is warranted.

There were several limitations to our study. First, GERD was diagnosed by endoscopic finding with mucosal injury. We retrospectively reviewed the result section for upper endoscopy of our electronic medical records. Moreover, we also diagnosed Barrett’s esophagus by endoscopic finding, not histologic confirmation. Second, since our study design was cross-sectional for the check-up cohort, there was a selection bias that included the individual’s food habits and socioeconomic status. Third, our cross-sectional study design made it hard to emphasize the temporal relationship between sarcopenia and the development of GERD. Fourth, despite current consensus definitions such as the European Working Group on Sarcopenia in Older People (EWGSOP), the European Society for Clinical Nutrition and Metabolism Special Interest Groups (ESPEN-SIG), and the International Working Group on Sarcopenia (IWGS) adopted muscle mass and function to define sarcopenia^3,37–39^, our study used only muscle mass. This was because our check-up center did not have an equipment to evaluate muscle function. In addition, routine program of most check-up centers only includes evaluation for muscle mass. Therefore, the data of our study may be enough worth predicting GERD in healthy subjects. Fifth, the patients’ information, such as combined DM, alcohol, smoking, and diet, were collected from a questionnaire. However, it is difficult to evaluate an exact amount of alcohol, frequency for smoking, and amount and concentration of food components. Moreover, taking acid suppressive drugs, such as proton pump inhibitor, is a confounding factor affecting GERD. However, questionnaire of our check-up center did not include history of medication for acid suppressive drug. Finally, because of the retrospective aspect of our study, we may have collected some incomplete data.

In conclusion, sarcopenia is associated with GERD. Moreover, sarcopenia and sarcopenic obesity are predictive factors for ERD. Our check-up center measured the patients’ anthropometric profile, which included body weight, height, and skeletal muscle mass. Therefore, our study is meaningful because the patients’ anthropometric measurements proved to be a useful data to predict GERD and ERD. Since the clinical presentations of sarcopenia are not specific, early detection by a routine health check-up is important. This study identified the association between sarcopenia and GERD, and proved our hypothesis. Further studies should be warranted to evaluate the mechanism for this relationship.
